# A comparative analysis of echocardiographic data for heart transplant eligibility in brain death cases

**DOI:** 10.1186/s43044-025-00709-0

**Published:** 2025-12-14

**Authors:** Azadeh Sadatnaseri, Marzieh Latifi, Habib Rahban, Andrew Boshara, Elahe Pourhosein, Abbas Soleimani, Shahrokh Karbalai, Mostafa Rouzitalab, Zahra Shajari, Saeeid Ghodsi, Sanaz Dehghani

**Affiliations:** 1 Department of Cardiology, Tehran University of Medical Sciences, Sina University Hospital, Tehran, Iran, Islamic Republic of; 2 Department of Public Health, School of Public Health and Safety, Shahid Beheshti University of Medical Sciences, Tehran, Iran, Islamic Republic of; 3 Medical Ethics and Law Research Center, Shahid Beheshti University of Medical Sciences, Tehran, Iran, Islamic Republic of; 4 Department of Cardiovascular Disease, Creighton University School of Medicine, St. Joseph Hospital and Medical Center, Phoenix, AZ USA; 5 Cardiovascular Research Foundation of Southern California, Beverly Hills, CA USA; 6 Creighton University School of Medicine, Dignity Health Cardiovascular Services, St. Joseph Hospital and Medical Center Phoenix, Phoenix, AZ USA; 7 Organ Procurement Unit, Sina University Hospital, Tehran University of Medical Sciences, Tehran, Iran, Islamic Republic of; 8 Iranian Tissue Bank & Research Center, Tehran University of Medical Sciences, Tehran, Iran, Islamic Republic of

**Keywords:** Heart transplant, Graft survival, Organ donation, Organ transplantation

## Abstract

**Background:**

The supply of donor hearts has fallen short of the increasing demand. This widening gap has led to a crisis marked by long waiting times and high mortality rates among those on waiting lists. This study aimed to compare the echocardiographic data of individuals whose hearts were eligible for transplant.

**Methods:**

This retrospective, cross-sectional study investigated all eligible brain-death cases conducted at Sina Hospital in Tehran, Iran. Based on echocardiographic data, patients were evaluated for heart donation (59 hearts that were deemed eligible vs. 39 hearts that were non-eligible for donation). The study used a custom checklist based on transthoracic echocardiography data. Data were analyzed using SPSS 18 software. A *P* < 0.05 was considered statistically significant.

**Result:**

The average age of the cases was 29.28 ± 10.59 years. Gender was not significantly associated with transplant eligibility (*P* = 0.46). In contrast, the cause of brain death was a significant factor (*P* = 0.04). There were statistically significant differences between the cause of brain death and left ventricular ejection fraction (LVEF) (F = 3.14, η² = 0.094, P = 0.029). No significant relationship was found between the cause of brain death and regional wall motion abnormality (RWMA) or pulmonary artery systolic pressure (PASP).

**Conclusion:**

Borderline cases should undergo reevaluation using repeat transthoracic echocardiogram or echocardiographic assessments of heart function with inotropic medications to increase the pool of potential heart donors due to limited organ donor options.

**Supplementary Information:**

The online version contains supplementary material available at 10.1186/s43044-025-00709-0.

## Introduction

Advanced Heart failure is a serious condition that requires urgent attention on a global scale [1]. Currently, an alarming 26 million individuals are affected by this life-threatening disease, placing significant burdens on patients, caregivers, and healthcare systems [2]. Statistics indicate that 2% of adults in developed countries suffer from heart failure, with an incidence rate of approximately 5–10 individuals per 1000 per year [3]. The prevalence of heart failure doubles with each decade of life, from under 1% for those under 40 to over 10% for those over 75 [4, 5]. 

Stage D Heart failure, as defined by the ACC/AHA, represents the final stage of irreversible cardiac dysfunction and includes etiologies such as ischemic, valvular, and congenital heart disease, as well as various other cardiomyopathies [1]. 

The progression of heart failure is associated with frequent hospitalizations due to decompensation, pump failure, lethal arrhythmias, and multiorgan failure [6]. 

Despite advancements in medical management and supportive care for end-stage heart failure patients, options like ventricular assist devices currently do not offer mortality benefits on the order of heart transplantations and present with elevated risks including bleeding, infections, stroke, right heart dysfunction, and pump thrombosis, making heart transplantation preferred for eligible individuals [7–9]. Improved survival rates and quality of life for heart transplant recipients in recent decades have highlighted the success of heart transplantation as a treatment option [9, 10]. However, the supply of donor hearts has fallen short of the increasing demand. This widening gap has led to a crisis marked by long waiting times and high mortality rates among those on waiting lists [11]. 

Although there has been a rise in overall heart transplants due to an increase of donors related to the opioid crisis, the imbalance between supply and demand has nevertheless worsened as a result of a decline in donor utilization alongside a surge in recipient needs [12]. The size of the donor pool remains the major limiting factor in heart transplantation [13]. Donor selection requires careful consideration of various factors [14], including urgent clinical and biochemical markers of the candidate, donor-recipient compatibility, and institutional risk tolerance [9]. 

Efforts have been made to improve the quality of organ preservation and minimize rejection criteria for potential donors to optimize the transplantation process [15]. Factors such as age, active malignancies, untreated infections, mental health issues, substance abuse, and other medical conditions must be assessed when determining the suitability of a brain-dead individual for organ donation [16]. 

In 2022, in Iran, only 130 heart transplants were performed, while there were 1630 patients on the waiting list. This mismatch between supply and demand has resulted in longer waiting times and increased mortality rates for patients in need of heart transplants. Various factors that influence the definition and suitability of ideal heart donors in Iran are outlined in Supplement 1.

This study aims to address the critical gap between donor heart availability and patient need by identifying specific echocardiographic parameters that can help distinguish between potentially suitable and unsuitable donor hearts. By collecting and analyzing echocardiographic data from brain death cases and classifying them into those eligible for heart donation versus those whose hearts were not suitable, we seek to establish evidence-based criteria that could potentially expand the donor pool without compromising transplant outcomes. The findings from this research will help clinicians make more informed decisions about donor heart eligibility, potentially reducing organ discard rates and improving access to transplantation for patients with end-stage heart failure.

## Patients and methods

This retrospective cross-sectional study analyzed all eligible cases of brain death, confirmed by advanced neurological assessments carried out at Sina Hospital at Tehran University of Medical Sciences from January 1, 2020, to September 30, 2022.

The inclusion criteria was donor age under 40 years if smokers and donors aged under 45 years old with normal angiography; performing echocardiography and ECG on the donor; testing for anti-Toxoplasma antibodies. Contraindications include uncontrolled systemic infection, poisoning with cyanide, or ecstasy, persistent ventricular arrhythmias, high doses of inotropes (dopamine, norepinephrine, or similar) despite adequate preload and afterload, significant wall motion abnormalities or low LVEF (< 50%), severe right ventricular dysfunction, LV hypertrophy (wall thickness ≥ 14 mm or ECG criteria), significant valve disease, significant coronary artery disease, or positive viral serology.

After documentation of brain death and obtaining authorization for organ donation, the donor was taken to the echocardiograph lab for further evaluation. Based on echocardiographic data, patients were evaluated for heart donation. Ninety-eight cases of brain death were categorized into two groups based on whether their hearts were transplanted or not (59 heart donors vs. 39 whose heart were not transplanted.

The study was approved by the research ethics committees of Sina Hospital, Tehran University of Medical Sciences (TUMS; Protocol no. IR.TUMS.SINAHOSPITAL.REC.1403.024), and was performed according to the principles of the Declaration of Helsinki.

The consent form for this study was signed by the next of kin or the legal guardian of the brain-dead persons prior to their examination. The signers were assured of the confidentiality of the information, and it was reaffirmed that the data would be used solely for research purposes with results generalized for publication.

This study utilized a demographic information questionnaire, which included age, gender, cause of brain death, and a researcher-developed checklist based on transthoracic echocardiographic data. Transthoracic echocardiography (TTE) was performed and stored using VIVID S60 (GE Healthcare) or VIVID E9 (GE Healthcare) equipment.

The echocardiograms primarily evaluated regional wall motion abnormality (RWMA), left ventricular ejection fraction (LVEF), and Interventricular septal thickness (Interventricular Septum) measured according to ASE guidelines [17]. 

In one case, transesophageal echocardiography was used due to poor TTE echo windows caused by subcutaneous emphysema. The patient was not excluded from the study as all measurements were within normal range. In some cases, the hearts were further assessed for the sum and rate of deformation of the myocardium through measurements of myocardial strain and strain rate, as determined by the echocardiographer [18]. 

All valves’ morphologies and performances were assessed using 2D, Color Doppler, pulse wave, and continuous wave studies. Pulmonary artery systolic pressure (PASP) was measured at the apical four-chamber and/or para sternal RV inflow and parasternal short axis view. PASP was estimated using the tricuspid regurgitant jet peak gradient (TRPG) and considering that all patients were under artificial ventilation, estimation of right atrial pressure based on the size and collapsibility of the IVC (inferior vena cava) through the subcostal view was ignored. In cases with a poor echo window or no tricuspid regurgitation (TR), the TRPG was not recorded. In patients with measurable pulmonary insufficiency (PI), the peak PI gradient (PG) was recorded. However, due to limited cases, it was only registered as normal or increased pulmonary artery pressure.

The donor’s hearts were evaluated in the supine position, under mechanical ventilation, and through all accessible window views. Two-dimensional and M-mode (MM) measurements were conducted to assess left ventricular (LV) size, including the thickness of the septum and the left ventricular posterior wall. Measurements of end-diastolic and end-systolic diameters were obtained using both parasternal long-axis and short-axis views.

These differing types of measurement were performed as each one provided different configurations of the imaging and echo window of the intubated patient.

LV systolic function, reflected as LVEF, was estimated using two-dimensional echocardiography with further assessment employing Simpson’s method (manual measurement of end diastolic and systolic volume by tracing endocardial borders) if the original measurements were inconclusive. Normal ranges for LVEF measured by two-dimensional echocardiography were considered to be 52% to 72% for men and 54% to 74% for women according to the American Society of Echocardiography and the European Association of Cardiovascular Imaging [17]. 

Strain studies were performed in cases of borderline LV systolic function. The data included dimensions and functions of the left and right chambers, LVEF, RWMA, diastolic function, valve performance, and PASP. Data was gathered bya cardiologist with at least 2 years’ experience of independent echocardiography or an echocardiologist; best quality loops were saved and sent through WhatsApp application to a cardiologist who was directly involved in heart transplantation center. And after accepting the heart’s function, the brain-dead heart went through the final stages of organ preparation and harvesting.

### Statistical analysis

Data analysis was performed using SPSS version 18. The normality of the continuous variables was assessed using the Kolmogorov-Smirnov test. For variables that were normally distributed, descriptive statistics were presented as mean and standard deviation. Categorical variables were reported as frequency and percentage. To compare the distributions of categorical variables between the two groups (those eligible for heart transplantation and those ineligible), Chi-square tests or Fisher’s exact tests were applied as appropriate.

To evaluate differences in echocardiographic criteria between the two groups, an independent *t* test was used for normally distributed continuous variables. Furthermore, a one-way ANOVA was performed to investigate the relationship between echocardiography data and the various causes of brain death.

A significance level of *P* < 0.05 was considered statistically significant for all analyses.

## Result

### Characteristics of recipients

Out of all potential donors, 59 (60.2%) were found to be eligible for heart donation, while 39 (39.8%) were rejected primarily due to low LVEF during the procurement echocardiographic assessment.

Out of all participants, 69 (70.4%) were male, with an average age of 29.3 ± 10.6 years. The main causes of brain death were head trauma (47; 49.5%) and drug intoxication (21; 22.1%).

The Mean ± SD left ventricular diastolic diameter and left ventricular diastolic posterior measurements in our participants were 43.14 ± 6.05 and 9.42 ± 1.77, respectively. Additional details have been included in Table 1.

**Table 1:** Demographic and clinical characteristics of the participants (*n* = 98)

	Heart donor (*n* = 59)	Non-Heart donor (*n* = 39)	Total (*n* = 98)	*P*
Age, years (mean)	28.22 ± 10.16	30.89 ± 11.15	29.28 ± 10.59	0.59^*^
LVDD (mm)	43.16 ± 6.73	43.10 ± 4.90	43.14 ± 6.05	0.98^*^
LVPW (mm)	9.35 ± 1.62	9.52 ± 1.99	9.42 ± 1.77	0.73^*^
IVSd (mm)	9.28 ± 1.61	9.21 ± 2.06	9.25 ± 1.79	0.75^*^
PASP (mmHg)	27.50 ± 6.25	29.47 ± 7.36	28.35 ± 6.78	0.001^*^
LVEF (%)	55.77 ± 4.46	47.08 ± 10.06	52.38 ± 8.30	0.001^*^
Sex				
Female	17 (28.8%)	12 (30.8%)	29 (29.6%)	0.46^**^
Male	42 (71.2%)	27 (69.2%)	69 (70.4%)	
Cause of brain death				
ICH	3 (5.1%)	8 (21.6%)	11 (11.6%)	0.04^***^
Head trauma	32 (54.23%)	15 (40.5%)	47 (49.5%)	
Drug intoxication	12 (20.33%)	9 (24.3%)	21 (22.1%)	
CVA	2 (3.39%)	1 (2.7%)	3 (3.2%)	
Tumor	2 (3.39%)	2 (5.4%)	4 (4.2%)	
Aneurysm	1 (1.69%)	1 (2.7%)	1 (1.1%)	
Hanging (suicide)	2 (3.39%)	0 (0%)	2 (2.1%)	
Others^+^	5 (8.48%)	1 (2. 7%)	6 (6.3%)	

*IVSd* Interventricular septum thickness (diameter per millimeter), *LVPW* Left ventricular posterior wall thickness (diameter per millimeter), *LVDD* Left ventricular diastolic diameter (diameter per millimeter), *LVEF* Left ventricular ejection fraction (per percent), *PASP* Pulmonary artery systolic pressure (per millimeter Hg)

*Independent Samples *t* Test; ** Fisher’s Exact Test; ***Chi-square Test

^+^Others included: Asthma, Gun shot, suicide (attempting suicide with a sharp object), seizure (status epilepticus), and Guillain–Barre syndrome

*P* < 0.05

Fisher’s Exact Test results showed no significant statistical difference between gender and the two groups related to heart transplant eligibility (*P* = 0.46).

Chi-square analysis revealed significant differences between the cause of brain death and the two groups related to heart transplant eligibility (*P* = 0.04). The cause of brain death may have an important influence on whether individuals in these groups are eligible for heart transplants (Table 1)

Results from a one-way ANOVA test indicate that there were statistically significant differences between the cause of brain death and LVEF (F = 3.14, η²= 0.094, *P* = 0.029). However, no significant differences were found between the cause of brain death and RWMA (F = 1.53, η²= 0.003, *P* = 0.97) as well as estimated PASP (F = 2.63, η²= 0.089, *P* = 0.055). See Table 2. The Tukey HSD results showed that head trauma group has the highest average LVEF (57.4792), while ICH group has the lowest (50.3125).

**Table 2:** Differences in echocardiography parameters across causes of brain death

	Sum of squares	df	Mean square	F	*P* value
LVEF					
Between groups	951.62	3	317.207	3.14	0.029
Within groups	9192.12	91	101.01		
PASP					
Between groups	341.720	3	113.907	2.63	0.055
Within groups	3498.46	81	43.19		
RWMA					
Between groups	0.036	3	0.012	0.79	0.97
Within groups	13.90	91	0.153		

*LVEF* Left ventricular ejection fraction, *PASP* Pulmonary artery systolic pressure, *RWMA* Regional wall motion abnormality

*P* < 0.05

The *t*-test results show significant differences in the mean LVEF between the two groups. with a *t* value of 5.76 indicating a strong effect (95% CI 5.69 to 11.69, *P* < 0.001).

Also, there is a significant difference in PASP between the two groups, with a *t* value of −3.46 indicating a strong effect (−3.46, 95% CI −7.76 to −2.10, *P* < 0.001).

The two groups had no significant differences in mean left ventricular size or left ventricular diastolic posterior, indicating these measurements may not be useful in distinguishing between the two groups regarding transplant eligibility.

Based on the data shown in Table 3, LVEF and PASP may be important indicators of eligibility for heart transplantation.

**Table 3:** Comparison of the mean ± SD of the constructs of the echo cardiology in two groups (heart eligible for transplantation or not)

	t	df	95% confidence interval of the difference		*P* value
			Lower	Upper	
LVEF	5.76	93	5.69	11.69	0.001^*^
LV Size	−1.44	92	−0.24	0.03	0.15
LVPW	−0.34	92	−0.86	0.61	0.73
PASP	−3.46	83	−7.76	−2.10	0.001^*^

*LVD Posterior* Left ventricular posterior wall thickness; *LVEF* Left ventricular ejection fraction; *LV size* Left ventricular (left ventricular size based on echocardiographer judgment normal, mild, moderate, or severe enlarged); *PASP* Pulmonary artery systolic pressure

*P < 0.05

## Discussion

In this study, the main cause of brain death in examined cases was head trauma. In general, our findings are the same as other studies that reported head trauma as the most common cause of brain death, especially in young drivers who have the highest risk of vehicular accidents [19]. 

In the present study, there is no significant difference between the cause of brain death and RWMA. Interestingly, the study showed significant differences between the cause of brain death and LVEF, based on our result head trauma group has the highest average LVEF, while ICH group has the lowest.

Heart failure due to adrenergic storm is a major cause for disqualification of the potential donors heart without a history of CVD [20]. According to the Donor Heart Selection Guideline in 2023, dobutamine stress echocardiography is performed in patients with LVEF < 45%. And reevaluate if the LVEF has increased during Dobutamine infusion. Improvement in LVEF can be predicted by ventricular wall augmentation [21]. 

Given the insufficient number of donors for heart transplants, it is crucial to be judicious and consider marginal organs in selected cases so as to optimize resource utilization and decrease wait list mortality. As long as such efforts do not reduce graft survival after transplantation.

Another interesting finding in our study was that the cause of brain death, gender, LVEF, LV size, LVDD, and LVPW were predictors of successful donor heart transplantation. Consistent with our findings, Khush et al. [22] declared several donor risk factors for allograft rejection based on previous literature. These included donor age, female sex, cerebrovascular accident (CVA)/stroke as the cause of death, underlying diseases, history of cocaine or methamphetamine use, high-inotrope requirement during donor management, cardiac troponin I >1.0 mg/L, left ventricular dysfunction (defined as left-ventricular ejection fraction < 50%), left-ventricular regional wall motion abnormalities, and left-ventricular hypertrophy. [22] There was a statistically significant difference between gender and LVEF (Preserved/Normal). Based on a meta-analysis by a global group in chronic heart failure, the risk of death appears to increase in patients with LVEF lower than 40% [23]. Heart failure with preserved ejection fraction (HFpEF) is common, especially among females [22, 23]. This aligns with previous studies connecting the use of female donor organs as a risk factor for death and rejection [24, 25]. 

Based on findings that initial left ventricular systolic dysfunction in potential heart donors is not rare, aligns with a growing body of several researches such as Oras et al. [16], which demonstrated, the transplantation of donor hearts with initial LVEF dysfunction is safe and yields recipient survival comparable to that of normal-functioning donor hearts. Similarly, the more recent study by Copeland et al. [16] reinforced this paradigm, showing that with appropriate management and selection of these “marginal” hearts can be successfully utilized to expand the donor pool without compromising transplant quality.

Normal PASP in eligible donors in this study is likely a reflection of a multifaceted approach to their care, such as utilizing lung-protective ventilation that minimizes intrathoracic pressure, employing judicious and targeted hemodynamic support (including inotropes when necessary but not excessively), and the inherent characteristic of this donor population: a general absence of significant pre-existing or acute conditions that would otherwise lead to increased pulmonary vascular resistance or right ventricular strain.

This study was conducted for the first time in Iran as one of the pioneer organ procurement statistics in the Middle East Society for Organ Donation (MESOT). Considering the lack of adequate organ availability in Iran, it may be possible to use marginal heart donors for marginal recipients, thereby decreasing the number of people on the waiting list.

## Limitation

There are several limitations to our study. This was a single-center retrospective study with a small sample size. The majority of donors were adolescents, and the application of our findings to pediatric donors may be limited. However, despite these limitations, our study is the first to evaluate the cardiovascular status of confirmed brain death cases and compare the results between procured and rejected cases in Iran.

## Conclusion

Transthoracic echocardiography is often a reliable equipment for evaluating donor heart suitability, with RWMA, decreased LVEF, and high PASP being key determinants for transplant ineligibility. LVEF and PASP are crucial, quantifiable metrics for refining heart donor selection protocols. While traditional cardiac risk factors and cause of brain death should guide the need for additional testing, successive reevaluation and consideration of borderline cases is recommended, especially given the limited organ availability and increased waitlist mortality against which most transplant programs contend.

Establishing clear, evidence-based thresholds for these values is essential. Donor management should be tailored to the cause of brain death, as it impacts cardiac function. Integrating these indicators into assessment algorithms optimizes donor eligibility, especially in resource-constrained systems.

## Supplementary Information

Supplementary Material 1.

## Data Availability

The datasets used and/or analyzed during this research study are available from the corresponding authors [Dr. Sanaz Dehghani with email: sanaz_dehghani2002@yahoo.com and Marzieh Latifi with email: marziye_latifi@yahoo.com] on reasonable request.

## Abbreviations

LVEF: Left ventricular ejection fraction
LVDD: Left ventricular diastolic diameter
LVPW: Left ventricular posterior wall thickness
RWMA: Regional wall motion abnormality
PASP: Pulmonary artery systolic pressure
IVC: Inferior vena cava
IVSd: Interventricular Septum
TR: Tricuspid regurgitation
TTE: Transthoracic echocardiography
MESOT: Middle East Society for Organ Transplantation
OPU: Organ procurement unit
