# Supplementary material for: A comparative analysis of echocardiographic data for heart transplant eligibility in brain death cases
Source: Egypt Heart J. 2025 Dec 14;77:111. doi: 10.1186/s43044-025-00709-0 (PMC12702817; doi:10.1186/s43044-025-00709-0)
Supplement: Supplementary file 1 — Supplementary Material 1. [file 43044_2025_709_MOESM1_ESM.docx]

| Supplement 1: Factors impacting the deﬁnition and usability of ideal heart donors (9) | |
| --- | --- |
| Factors | **Details** |
| Age | <40 years. |
| No history of chest trauma |  |
| No history of cardiac disease |  |
| No prolonged hypotension or hypoxemia during pre-harvest time |  |
| Appropriate hemodynamics |  |
| Mean arterial pressure | > 60 mmHg |
| Central venous pressure | Between 8 - 12 mmHg |
| Inotropic support (dopamine or dobutamine) | Less than 10 µg/kg/min |
| Normal electrocardiogram |  |
| Normal echocardiogram |  |
| Normal cardiac angiography (if indicated by donor age and history) |  |
| Negative serology (hepatitis B surface antigen, hepatitis C virus, HIV) |  |

^*Adopted from Shakerian B, Sadatnaseri A. et al. The outcomes of marginal donor hearts compared with ideal donors: a single-center experience in Iran. Korean Journal of Transplantation. 2022;36(2):136-142.^
